# Association of Cesarean Birth with Body Mass Index Trajectories in Adolescence

**DOI:** 10.3390/ijerph17062003

**Published:** 2020-03-18

**Authors:** Yunping Zhou, Yanqing Zhang, Yun Sun, Dongfeng Zhang

**Affiliations:** 1 School of Nursing, Qingdao University, Qingdao 266071, China; lwzhouyunping@163.com; 2 Zibo Center for Disease Control and Prevention, Zibo 255026, China; yanqing089@126.com; 3 Friedman School of Nutrition Science and Policy, Tufts University, Boston, MA 02111, USA; yun.sun@tufts.edu; 4 School of Public Health, Qingdao University, Qingdao 266071, China

**Keywords:** delivery mode, obesity trajectory, adolescence

## Abstract

*Background:* This study aimed to identify patterns of body mass index (BMI) changes in adolescence and to assess whether delivery mode (Cesarean and vaginal delivery) was associated with BMI trajectories. *Methods:* This study was conducted among 569 adolescents aged 10–15 years that resided in the city of Zibo, China. The height and weight of each participant were repeatedly measured at 10, 11, 12, 13, 14 and 15 years. Group based trajectory modeling (GBTM) was used to estimate BMI change trajectories, and multinomial logistic regression was conducted to evaluate the independent association of delivery mode and BMI trajectory classes. *Results:* Of the 569 participants, 407 (71.5%) were vaginal deliveries and 162 (28.5%) were Cesarean deliveries. Five distinct long-term BMI trajectories were identified: “persistent healthy weight” (57.5%), “persistent underweight” (6.5%), “obesity to healthy weight” (7.8%), “progressive overweight” (10.6%), “progressive obesity” (17.6%). Adjusted multinomial logistic models revealed a twofold increase in risks between ages 10–15 years of “progressive obesity” trajectory (OR = 2.50, 95% CI: 1.42, 4.41) for children born through Cesarean section compared with vaginal birth. *Conclusions*: Five distinct long-term BMI trajectories were identified during adolescence in our research, and we confirmed that Cesarean birth was significantly increased the risk of “progressive obesity” trajectory but not the “obesity to healthy weight” trajectory.

## 1. Introduction

Prevalence of overweight and obesity has substantially increased among children and adolescence worldwide [1,2]. Overweight and obesity in childhood and early adulthood are known to have immediate and long-term health consequences and are now recognized important public health concerns [3,4]. Although considerable cross-sectional and longitudinal research have evaluated the underlying risk factors of obesity, the mechanisms are complex and still have remained uncertain. There was a growing body of evidence suggesting that children born by Cesarean experience higher rates of adverse health outcomes, including obesity later in life [5,6]. It was speculated that infants born vaginally developed a gut microbiome that differs from Cesarean birth [7,8], which might influence energy regulation and contribute to the development of obesity later in life. In addition, altered postnatal feeding in born infants via cesarean delivery vs. those delivered vaginally may have long-term effects on appetite regulation or energy metabolism that may cause the significant increase in childhood obesity [9].

Recently, China has witnessed a rapid increase of Cesarean section rates in recent years. A more recent study reported that the average Cesarean section rate increased from 28.8% in 2008 to 34.9% in 2014 [10]. Given the high Cesarean section rates and potential importance of prenatal influences on childhood and adolescence obesity, there is a need to explore the relationship of Cesarean section and Body Mass Index (BMI) developmental trajectories in children and adolescence [11,12]. BMI trajectories reflected the potential obesity dynamic changing patterns during the life course [13]. Such research will enhance our understanding to identify different etiologic pathways of obesity onset and development during adolescence. Moreover, obesity in adolescence is a key predictor of obesity in adulthood; this time frame represents an essential window to prevent lifelong weight and the related health issues [14].

To our knowledge, no study has reported on how the mode of delivery was related to the subsequent group-based trajectories of body shape in adolescence. The objective of the current study was to identify distinct BMI trajectories during adolescence with measures of BMI at various points in time, and to evaluate associations of delivery mode with offspring BMI trajectories in adolescence. Improved understanding of the role of obesity/BMI in posture development is needed for improved prevention and management strategies.

## 2. Methods

### 2.1. Population

Height and weight data in this study came from the National Surveys on Chinese Students’ Constitution and Health, which was an ongoing nationally representative school-based sample of children followed into adolescents [15]. This study used a multistage, stratified, clustered sampling design. A sample of two schools from Zibo city were selected to ensure the sample was representative in terms of urbanicity, school size and school type. The participants in this retrospective study was conducted among 621 children aged 10 years old in 2013. They were followed up at ages of 11, 12, 13, 14 and 15 years to obtain information of height and weight. The characteristic data were acquired by questionnaires from the participants’ mothers for the first contact when the children were 10 years old (baseline). The baseline information mainly included sex, birth weight, delivery mode, gestational age, children’s history of hypertension, maternal pregnancy age, maternal BMI status (baseline), maternal schooling (baseline) and household income (baseline). A validation study [16] showed that long-term maternal recall of many events associated with pregnancy, including diagnosis of major complications (hypertensive disorders of pregnancy, gestational diabetes and placenta previa), offspring birth weight, gestational age at delivery and pregnancy multiplicity, were highly reproducible and specific. After removing participants who had missing data on exposure variables (n = 52), a total of 569 participants (91.62% of eligible sample) were included in the study sample. Informed consent was obtained from parents, and study participants gave their consent when applicable. The study was conducted in accordance with the Declaration of Helsinki, and the protocol was approved by the Ethics Committee of the Affiliated Hospital of Qingdao University (Project identification code: 20130817).

### 2.2. Outcomes

All technicians were required to pass a standard training course for anthropometric measurements. Height was measured using a wall-mounted stadiometer to the nearest 0.1 cm, and weight was measured with a standardized scale to the nearest 0.1 kg. Both height and weight were measured twice, and the mean value was recorded. Body mass index (BMI) was calculated using the following formula: BMI = weight (kg)/height^2^ (m^2^). Subjects were defined as being four body shape categories: underweight, healthy weight, overweight and obesity by referring to the age-specific and sex-specific classification criteria for Screening Overweight and Obesity (WS/T586-2018) in Chinese Children and Adolescents, the standard was released by the Chinese National Health and Family Planning Commission in 2018 (Supplementary Table S1) [17].

### 2.3. Statistical Methods

We used a group-based trajectory modeling (GBTM) approach implemented in SAS Proc Traj (SAS Institute, Inc., Cary, North Carolina, CA, USA) to identify trajectory groups that shared similar underlying trajectories of body shape [18]. This method is designed to identify relatively homogeneous clusters of developmental trajectories within the population and has been successfully applied in large prospective cohort studies [19]. Participants were assigned to one of the trajectories to which they had the highest estimated probability of belonging.

Models were estimated with two to six groups, while the testing parameter estimates for linear and quadratic terms. The previous exploratory steps were performed for boys and girls separately, and no differences in the number of groups or trajectory shapes compared with the model that included both sexes. Thus, we modeled the trajectories with boys and girls in the same model. The choice of the best model was based on the results from the trajectory analyses and the interpretability of the groups, different indices of goodness of fit and discrimination (Bayesian information criteria (BIC), log-likelihood, proportion of subjects classified in each class with a posterior probability >0.7 and values of mean posterior class membership probabilities) as well as clinical plausibility [20].

Odds ratios (OR) and their associated 95% confidence intervals (CIs) were calculated using multinomial logistic regression analysis, which was applied to estimate associations between delivery mode (vaginal vs. Cesarean) and BMI trajectories (outcome variable; “persistent healthy weight” trajectory formed the reference category). Two models were tested: the first crude model, whereas the second model controlled for the following potential confounders: sex (boys vs. girls), birth weight (low birth weight < 2500 g; normal birth weight, 2500-4000 g; high birth weight, ≥4000 g), gestational age at delivery (continuous), children’s history of hypertension (yes vs. no), baseline maternal BMI (BMI ≤ 18, 18–24, ≥24 kg/m^2^), maternal age (continuous), maternal schooling (<9 years vs. ≥9 years) and household income(<60,000 ¥ per year, ≥60,000 ¥ per year). All statistical analyses were conducted using SAS V.9.3 (SAS Institute, Cary, North Carolina, CA, USA). The statistical significance level was set to α = 0.05 for all association analyses.

## 3. Results

### 3.1. Body Shape Trajectories

**Figure 1:**
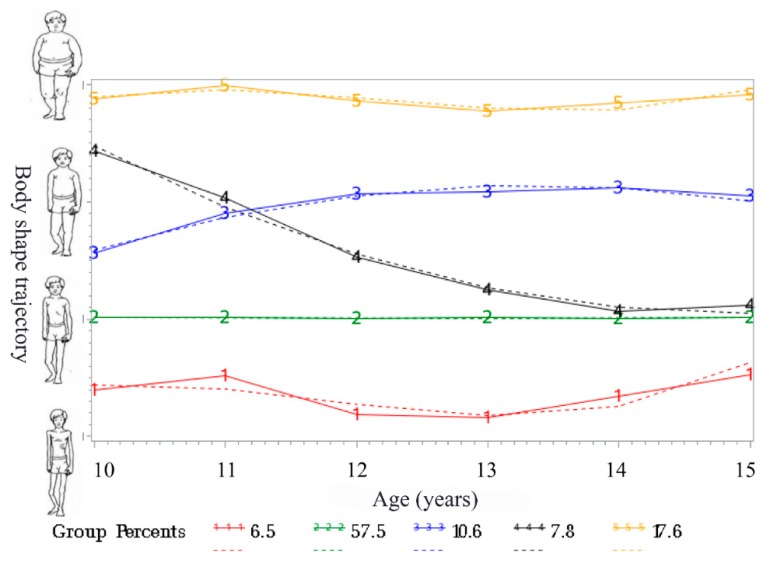
Trajectories of body shape during adolescence from 10–15 years. Solid lines represent observed values, and dashed lines represent expected values. Group 1, ‘‘persistent underweight” (6.5%); Group 2, ‘‘persistent healthy weight’’ (57.5%); Group 3, ‘‘progressive overweight’’ (10.6%); Group 4, ‘‘obesity to healthy weight’’ (7.8%); Group 5, ‘‘progressive obesity” (17.6%).

Using BIC to assess the goodness-of-fit of the competing GBTM models, we identified five discrete BMI trajectories among the 569 participants (Figure 1, Table 1). The 6.5% followed a trajectory where the average predicted BMI levels remained within underweight body shape throughout follow-up (“persistent underweight” group, Trajectory 1, n = 37), 57.5% of adolescents maintained healthy body shape (“persistent healthy weight”, Trajectory 2, n = 327); 10.6% of participants maintained progressive overweight body shape (Trajectory 3, “progressive overweight”, n = 62); 7.8% started obese then experienced a decrease in body shape (Trajectory 4, “obesity to healthy weight,” n = 43); 17.6% followed a trajectory of progressive obese body shape (Trajectory 5, “progressive obesity,” n = 100). 

We identified disparities in the proportions and the patterns of the BMI trajectories modeled separately by sex, delivery mode and mother education (*p* < 0.05) (Table 1). Compared to “persistent healthy weight” trajectory, boys were more likely to follow a trajectory of progressive overweight and obesity and persistent underweight.

### 3.2. Association of Delivery Mode with Body Shape Trajectory Groups

In Table 2, we show unadjusted and multivariable-adjusted associations of delivery mode with the body shape trajectories in adolescence. The “persistent healthy weight” trajectory was selected as reference groups in the generalized linear regression analyses for both boys and girls. Before adjustment for potential confounders, Cesarean section delivery compared to vaginal delivery was associated with 2.41 (95% CI: 1.51, 3.85) times greater odds of the “progressive obesity” trajectory compared with the “persistent healthy weight” trajectory. This association was slightly attenuated, but remained statistically significant (OR = 2.50, 95% CI: 1.42, 4.41) after adjustment for potential confounders. The significant positive relationship between Cesarean section and the risk of “progressive obesity” trajectory was also found among boys (OR = 2.42, 95% CI: 1.10, 5.33) and girls (OR = 2.38, 95% CI: 1.01, 5.62), respectively.

No significant associations between Cesarean delivery and the “persistent underweight” (OR = 1.06, 95% CI: 0.42, 2.68), “progressive overweight” (OR = 1.11, 95% CI: 0.55, 2.27), and “obesity to healthy weight” (OR = 0.72, 95% CI: 0.27, 1.88) trajectories compared with the “persistent healthy weight” trajectory were observed after adjusting for potential confounders.

## 4. Discussion

In this study, five BMI trajectories that spanned 10–15 years during adolescence were identified. Our study suggested that Cesarean birth was associated with a 150% increase in the risk of offspring “progressive obesity” trajectory during adolescence compared with the “persistent healthy weight” trajectory after adjusting for major confounding factors, and the relationship remained consistent across strata of sex. The observations of this study provided new insights into origins of delivery mode-related obesity in early adult life and emphasized the importance of Cesarean birth for assessing the level-independent BMI trajectories during adolescence. Moreover, our study also confirmed that Cesarean birth was only positively associated with adolescents following the “progressive obesity” trajectory, and not the “obesity to healthy weight” trajectory.

The patterns and validity of these trajectories in our study was supported by their similarity to trajectories reported in previous studies with different approaches and samples [21,22,23]. For example, Buscot et al. noted six distinct child-to-adult BMI trajectories and indicated that trajectories reach or persist at high levels associate with CVD risk factors in adulthood [24]. Moreover, four (high-rising, low-to-high, median-stable and low-stable) children’s BMI patterns were identified in a study conducted with the National Institute of Child Health and Human Development’s Study of Early Child Care and Youth Development (SECCYD) in the USA [25]. Enhanced knowledge of the dynamics of BMI change across the early life course, especially the development of pathologic versus healthy age-related BMI trajectories, might help guide clinical and public health practice by suggesting critical and sensitive windows for intervention targeted at reducing the incidence of adult obesity with the aim of halting the progression of existing obesity [26].

Although numerous studies have evaluated the relationship of Cesarean delivery and offspring obesity, the results were inconsistent. Several studies indicated that Cesarean delivery increased the risk of offspring obesity, while others did not [6,27]. Given that the studies were conducted in different countries, with participants born in different time periods and followed for different durations, with various strategies used to control confounding. Most previous studies focused on BMI in a single or limited number of measurements, ignoring the dynamic BMI changes during the life course. Tracking trajectory patterns over time during adolescence accounted for dynamic changes and provided an important insight for further consideration [28]. Proposed mechanisms explaining the observed association between CS and subsequent obesity in the offspring include, but are not limited to, hormonal surge, stress exposure [9], DNA methylation [29] and microflora transmission (hygiene hypothesis). Early feeding patterns and a lower rate of breastfeeding initiation and shorter breastfeeding duration are known to be consequences of cesarean delivery and may play a role in the biological pathway between cesarean delivery and later-life obesity. In a study performed in childhood, adolescence and early adulthood from three birth-cohort studies in Southern Brazil [30], the researchers indicated that Cesarean sections did not seem to increase the risk of obesity during childhood, adolescence or early adulthood. In our study, the associations among Cesarean sections and different BMI trajectories varied by sex. There were positive relationships between Cesarean birth and different BMI trajectories compared with the “persistent healthy weight” trajectory in girls, while the associations were mixed in boys. The underlying mechanisms might be due to unadjusted confounding such as lifestyle and physical activity. Future studies are warranted to examine the observed sex difference. In addition, children and adolescents, a period of rapid growth with a greater amount of activity and development of physical functions, the stability of weight status was relatively low [31]. The measurement of obesity in the above-mentioned study was on a single time point, which ignored the dynamic of obesity in childhood and adolescence. In our research, the two groups, “progressive obesity” trajectory and “obesity to healthy weight” trajectory, differed in the association with Cesarean delivery, despite their similarly higher weight status at the beginning of the study. This might explain some studies did not find positive relationship between Cesarean birth and obesity [32]. Therefore, a better understanding of the trajectories of those who develop obesity or those who had high BMI in adolescence but did not become obese in late adolescence was particularly important from a public health perspective.

The current study had multiple strengths and was able to address the most salient limitations of previous studies. Our findings extended and refined evidence in the association between delivery mode and offspring obesity trajectories in adolescence. The cohort study design, long-term follow-up and the use of GBTM allowed us to examine the relation of Cesarean section and offspring obesity risk during adolescence and to provide precise estimates of the association. Parameters of GBTM included the probability of membership in a particular latent trajectory class for each individual, as well as the intercept and slope of the trajectory for each class [19].

There were limitations of the current study that merit mention. First, the most important limitation of our study was that we lacked data on intra-partum indications for Cesarean delivery. However, findings from studies in which subgroup analyses were performed by the indication for Caesarean section [33] or whether Caesarean section was done before or after the onset of labor [34] did not show significant differences between the groups. Nevertheless, further studies are needed to delineate whether the “progressive obesity” trajectory risk associated with Cesarean delivery depends on the determinate factors for Cesarean birth. Secondly, the information of delivery mode and birth weight were recalled by mothers. However, our investigators helped mothers recall the information carefully, and confirmed this information with the fathers if necessary. Thirdly, as this was an observational study, we cannot rule out the possibility for residual or unmeasured confounding. Limited information was obtained about puberty status from the participants, there might be significant hormonal changes that could impact body size and might be related to mode of delivery over the age range of 10–15 years. Moreover, we were also unable to get information on breastfeeding and the father’s BMI, which might be a potential confounder for the association of Cesarean delivery and BMI trajectories. Some research indicated that children who were breastfed might have a lower risk of childhood obesity. However, other studies suggested that any observed protective effect of breastfeeding on the BMI may be due to unadjusted confounding [35]. The human milk metabolome varied by maternal obesity [36] and might be mediated by the association between Cesarean delivery and BMI trajectories. Further studies are needed to clarify this association.

## 5. Conclusions

In summary, our findings supported the importance of long-term tracking of children’s BMI. We found five heterogeneous trajectories of BMI development and indicated that Cesarean (vs. vaginal) delivery increased the risk of “progressive obesity” trajectory and not the “obesity to healthy weight” trajectory during adolescence. Identifying and intervening with children at risk for rapidly rising and “progressive obesity” trajectories should be the focus of obesity prevention efforts for the highest value to public health.

## Figures and Tables

**Table 1:** Participant characteristics for each of the five different body mass index trajectory groups.

Variable	N (% of Sample)	Persistent Underweight (Trajectory 1)	Persistent Healthy Weight (Trajectory 2)	Progressive Overweight (Trajectory 3)	Obesity to Healthy Weight (Trajectory 4)	Progressive Obesity (Trajectory 5)	*p* ^a^
**Child characteristics**							
N	569	37	327	62	43	100	
Sex							<0.001
Girls (%)	294 (52.3%)	13 (35.1%)	195 (59.6%)	27 (43.5%)	19 (44.2%)	40 (40.0%)	
Boys (%)	275 (47.7%)	24 (64.9%)	132 (40.4%)	35 (56.5%)	24 (55.8%)	60 (60.0%)	
Delivery mode (%)							0.003
Vaginal (%)	407 (71.7%)	29 (78.4%)	243 (75.4%)	44 (70.5%)	33 (76.7%)	58 (56.0%)	
Cesarean (%)	162 (28.3%)	8 (21.6%)	80 (24.6%)	19 (29.5%)	10 (23.3%)	45 (44.0%)	
Gestational age at birth (weeks, mean (SD))	38.9 (1.4)	39 (0.9)	38.9 (1.5)	38.9 (1.5)	38.8 (1.4)	38.9 (1.4)	0.699
Birth weight (%)							0.519
LBW	24 (4.5%)	4 (11.1%)	13 (4.2%)	1 (1.6%)	1 (2.5%)	5 (5.3%)	
NBW	415 (77.1%)	25 (69.5%)	240 (78.4%)	48 (78.7%)	33 (82.5%)	69 (72.6%)	
HBW	99 (18.4%)	7 (19.4%)	53 (17.3%)	12 (19.7%)	6 (15.0%)	21 (22.1%)	
History of hypertension (%)							0.039
Yes	235 (42.9%)	14 (38.9%)	125 (39.4%)	27 (45.8%)	14 (36.8%)	55 (56.2%)	
No	313 (57.1%)	22 (61.1%)	192 (60.6%)	32 (54.2%)	24 (62.2%)	43 (43.8%)	
Household income (per year)							
<60,000 ¥	259 (46.5%)	16 (44.4%)	148 (46.3%)	23 (37.7%)	20 (46.5%)	52 (53.6%)	0.415
≥60,000 ¥	298(53.5%)	20 (55.6%)	172 (53.7)	38 (62.3%)	23 (53.5%)	45 (46.4%)	
**Maternal characteristics**							
Maternal pregnancy age (y, mean (SD))	26.5 (2.9)	26.8 (2.9)	26.5 (2.6)	25.6 (3.6)	27.1 (3.2)	26.5 (3.1)	
Mother schooling (%)							0.007
≤9 years	272 (47.8%)	17 (45.9%)	158 (48.3%)	36 (58.1%)	27 (62.8%)	34 (39.1%)	
>9 years	297 (52.2%)	20 (54.1%)	169 (51.7%)	26 (41.9%)	16 (37.2%)	53 (60.9%)	
Maternal BMI status (%)							0.289
Underweight	30 (5.5%)	1 (2.9%)	17 (5.5%)	4 (6.9%)	4 (9.8%)	4 (4.1%)	
Healthy weight	349 (64.3%)	25 (71.4%)	201 (64.6%)	35 (60.3%)	31 (75.6%)	57 (58.2%)	
Overweight/Obesity	164 (30.2%)	9 (25.7%)	93 (29.9%)	19 (32.8%)	6 (14.6%)	37 (37.7%)	

LBW, low birth weight, <2500 g; NBW, normal birth weight, 2500–4000 g; HBW, high birth weight, ≥4000 g; BMI, body mass index. ^a^ *p*-values from ANOVA F-tests (comparisons of means) and from x^2^ tests of independence (comparison of proportions).

**Table 2:** Crude and multivariable-adjusted odds ratios (OR) and 95% confidence intervals (CI) for offspring obesity trajectories associated with Cesarean vs. vaginal delivery.

Variable	Trajectory 1 vs. Trajectory 2		Trajectory 3 vs. Trajectory 2		Trajectory 4 vs. Trajectory 2		Trajectory 5 vs. Trajectory 2	
	OR	95% CI	OR	95% CI	OR	95% CI	OR	95% CI
Both sexes								
Vaginal delivery	1.00	-	1.00	-	1.00	-	1.00	-
Cesarean delivery ^1^	0.85	0.37–1.92	1.28	0.70–2.35	0.93	0.44–1.97	2.41	1.51–3.85
Cesarean delivery ^2^	1.06	0.42–2.68	1.11	0.55–2.27	0.72	0.27–1.88	2.50	1.42–4.41
Boys								
Vaginal delivery	1.00	-	1.00	-	1.00	-	1.00	-
Cesarean delivery ^1^	0.61	0.19–1.91	1.21	0.53–2.79	0.61	0.19–1.91	2.32	1.21–4.43
Cesarean delivery ^2^	0.53	0.13–2.19	1.08	0.38–3.05	0.35	0.06–1.87	2.42	1.10-5.33
Girls								
Vaginal delivery	1.00	-	1.00	-	1.00	-	1.00	-
Cesarean delivery ^1^	1.37	0.40–4.66	1.37	0.56–3.36	1.42	0.51–3.96	2.52	1.25–5.11
Cesarean delivery ^2^	2.42	0.61–9.56	1.26	0.46–3.44	1.17	0.33–4.14	2.38	1.01–5.62

^1^ Model 1 was the crude model; ^2^ Model 2 was adjusted for sex, birth weight (LBW, low birth weight, <2500 g; NBW, normal birth weight, 2500-4000 g; HBW, high birth weight, >4001 g), gestational age, maternal pregnancy age, maternal baseline body mass index status (BMI ≤ 18, 18–24, ≥24 kg/m^2^), maternal schooling (baseline), offspring history of hypertension, household income and school variable.

## Supplementary Materials

The following are available online at https://www.mdpi.com/1660-4601/17/6/2003/s1, Table S1: BMI cutoff points by age and sex in Chinese school-aged children (6–18 years).
