# Supplementary material for: Association of Cesarean Birth with Body Mass Index Trajectories in Adolescence
Source: Int J Environ Res Public Health. 2020 Mar 18;17(6):2003. doi: 10.3390/ijerph17062003 (PMC7142989; doi:10.3390/ijerph17062003)
Supplement: Supplementary file 1 [file ijerph-17-02003-s001.pdf]

**Table S1.** BMI cutoff points by age and sex in Chinese school-aged children (6–18 years).

| Age (Years) | Boys | | Girls | |
|---|---|---|---|---|
| | **Overweight** | **Obesity** | **Overweight** | **Obesity** |
| 6.0- | 16.4 | 17.7 | 16.2 | 17.5 |
| 6.5- | 16.7 | 18.1 | 16.5 | 18.0 |
| 7.0- | 17.0 | 18.7 | 16.8 | 18.5 |
| 7.5- | 17.4 | 19.2 | 17.2 | 19.0 |
| 8.0- | 17.8 | 19.7 | 17.6 | 19.4 |
| 8.5- | 18.1 | 20.3 | 18.1 | 19.9 |
| 9.0- | 18.5 | 20.8 | 18.5 | 20.4 |
| 9.5- | 18.9 | 21.4 | 19.0 | 21.0 |
| 10.0- | 19.2 | 21.9 | 19.5 | 21.5 |
| 10.5- | 19.6 | 22.5 | 20.0 | 22.1 |
| 11.0- | 19.9 | 23.0 | 20.5 | 22.7 |
| 11.5- | 20.3 | 23.6 | 21.1 | 23.3 |
| 12.0- | 20.7 | 24.1 | 21.5 | 23.9 |
| 12.5- | 21.0 | 24.7 | 21.9 | 24.5 |
| 13.0- | 21.4 | 25.2 | 22.2 | 25.0 |
| 13.5- | 21.9 | 25.7 | 22.6 | 25.6 |
| 14.0- | 22.3 | 26.1 | 22.8 | 25.9 |
| 14.5- | 22.6 | 26.4 | 23.0 | 26.3 |
| 15.0- | 22.9 | 26.6 | 23.2 | 26.6 |
| 15.5- | 23.1 | 26.9 | 23.4 | 26.9 |
| 16.0- | 23.3 | 27.1 | 23.6 | 27.1 |
| 16.5- | 23.5 | 27.4 | 23.7 | 27.4 |
| 17.0- | 23.7 | 27.6 | 23.8 | 27.6 |
| 17.5- | 23.8 | 27.8 | 23.9 | 27.8 |
| 18.0- | 24.0 | 28.0 | 24.0 | 28.0 |

Source: This standard was released by the Chinese National Health and Family Planning Commission in 2018.
